# Supplementary material for: Salivary concentrations of secretory leukocyte protease inhibitor and matrix metallopeptidase-9 following a single bout of exercise are associated with intensity and hydration status
Source: PLoS One. 2023 Nov 22;18(11):e0291297. doi: 10.1371/journal.pone.0291297 (PMC10664895; doi:10.1371/journal.pone.0291297)
Supplement: Attachment — Submitted filename: Review PONE-D-22-17078.docx [file pone.0291297.s001.docx]

Review PONE-D-22-17078

General comments:

Thanks for the opportunity to review this work. The authors present a study in which healthy individuals undertook one rest and four exercise protocols to investigate a relatively broad spectrum of immune/inflammatory biomarker responses to four exercise protocols.

Having referred to the other papers arising from this design, it appears the study has been conducted to a high standard and generates useful data that will contribute novel observations to the field. However, I do not fully understand the rationale for slicing the work into multiple publications. Much of the important information required to interpret the present findings has already been published (e.g. subject characteristics; Borg RPE; hydration status as well as known immune markers such as immune cell counts). This means that this paper reads with a lot of seemingly missing data that has already been published. I understand the authors were probably striving to avoid double publication of results, but repetition of certain details in my opinion is essential in order provide the full picture for the reader. For example, the glutamine bolus administered in Kartaram (2019) is omitted completely in this paper – could this modulate immune biomarkers and thus be of importance to the reader? In fact, I would say the findings here complement the findings of the paper of Kartaram (2020) well and it is a shame to see them separated out. Sometimes the value of the sum of the parts is lesser than the whole.

However, it is my role as a reviewer to give recommendations to the authors as to how this specific manuscript could be improved. I do believe the data present novel value and that the manuscript has potential for publication. Nevertheless I have a relatively long list further comments that I hope the authors find constructive and will consider should they choose to revise the manuscript.

Twenty two inflammatory markers were measured in saliva and/or serum but the whole rationale and abstract focuses on just two – SLPI and MMP-9. Were these main aims established a priori or post-hoc based on the results? Please be clear throughout the manuscript on what were the main (a priori) aims and hypothesis as well as secondary aims and any exploratory or post-hoc analyses. It should help to then relate aspects of e.g. design choices and the weight given to particular results/conclusions back to the aims.

The statistical analyses are described in careful detail but then the results lack transparency with a lack of p values for specific fixed factors and interactions as well as a lack of raw and supporting data.

The rigour of the scientific writing could do with additional review, to ensure precision of terms and arguments and that references are provided where appropriate. In addition, the results must be reported to a much greater degree of specificity. There are also a handful of small grammatical errors and quirks in the text and thus it would benefit from a final review for clarity as well as correctness.

Specific comments:

**Abstract**

L35: See above comment - was the aim to investigate just these two proteins (SLPI and MMP-9) or a whole panel of 22 markers (of which these two came out as significant)?

L38: ”exercise protocols of 1 hour duration in a randomly assigned cross-over design of their individual Wmax” – this reads a bit strangely as the *design* isn’t related to Wmax – perhaps “based on their individual Wmax” or consider beginning a new sentence to highlight the individual protocols/intensities?

L40: can you include the specific time-points for saliva sampling?

L44: “Values” of what? Please be specific.

L46: Conclusions – It is a bit of a jump to go from an association with intensity and hydration status to a biomarker of immunity in general. Please consider carefully the specific conclusions that can be drawn from the observations in the present study.

L47: the exercise is described as “strenuous” but is this descriptor appropriate for 1 h at e.g. 50% Wmax?

**Introduction**

I miss a specific rationale for how SLPI and MMP-9 secretion in saliva would be hypothesized to be affected by exercise, given what is known about other immune responses to exercise. What makes you think that these markers might be associated with exercise load – or are they simply important aspects of functional immunity that haven’t been previously measured?

L51 – Please include a reference for the first sentence if possible.

L52 – I am curious as to any examples of existing biomarkers that can be provided here that reliably and accurately monitor “stress load”. It appears to me that although the field has been looking for such markers for some time, none (especially not in saliva) have really achieved this yet – at least not for individual monitoring and decision-making.

L54 – Reference 1 (Pedersen et al) is a relatively old study that I am not sure accurately supports the statement made in this sentence.

L54-55 – “Exercise has multiple effects on the immune system” – is a quite vague statement and I wonder if you can start by being any more specific?

L56 – “…strenuous exercise can temporarily impair both the innate and the adaptive immune system” – this assumption has been contended in recent years (see e.g. Campbell and Turner 2018 Front Immunol; Simpson et al 2020 Ex Immunol Rev). Once again, it would be helpful to be more specific about the contexts of “strenuous” exercise that have been shown to disturb immune function – exercise can be characterized by several other prescription factors (e.g. intensity, duration, modality, continuous/intermittent) and I suspect “strenuous” may not be sufficiently specific to describe the relationship between exercise and immunity. As the authors note in the following paragraph, exercise-induced responses are dependent on intensity, duration and hydration (among other things – I would contend also modality and intermittent/continuous prescriptions are important factors).

L58 – sentence beginning “In case” needs a reference.

L61 – “immune distortion” – correct terminology?

L61 – “increased systemic inflammatory “state” – can a “state” be increased?

L61 – what “important roles”?

L64 – sentence beginning “Exercise-induced responses” needs a reference as well as the following sentence. In addition, the whole paragraph that follows could benefit from specific reference to these prescription factors (intensity, duration and hydration status) when presenting what is known about exercise-induced immune responses.

L66 – if the plasma concentration of both pro and anti-inflammatory mediators increases, what does this mean for ‘net’ inflammation?

L77 – “aspects” 🡪 “factors”?

L80 – “find new markers” – of what, specifically?

L81 – “it is increasingly acknowledged that saliva ~~often~~  can provide~~s~~ a useful matrix to study biomarkers of general health, stress and other variables”. This sentence could do with a reference, but I would contend that a) use of salivary biomarkers is not new – for example salivary IgA was measured in 1982 by Tomasi in cross-country skiers and b) saliva as a matrix has also been relatively frequently criticized as highly variable and susceptible to confounding factors, as the authors themselves note in the discussion. Jos Bosch provides an excellent review of some additional confounding mechanisms for salivary biomarkers in his chapter in Saliva Monographs in Oral Science (2014) – available here: [10.1159/000358864](https://pubmed.ncbi.nlm.nih.gov/24862598/). Consideration of the secretory mechanisms of the novel proteins presented here might benefit how their responses to exercise can be interpreted.

L100-105 – It is unclear how the statements about other heterogeneous diseases (CV disease, periodontitis, cancer and IBD) and the further discussion about the role of MMP-9 and IBD is relevant in relation to your hypothesis and research question.

L109 – the aim should be stated clearly, ideally accompanied by a hypothesis, that in turn is substantiated with further specific background to inform the research question.

**Methods**

“Subjects and Methods” seems like an unusual header for this section, I would imagine “methods” would suffice unless specified by the journal.

Please report at minimum age, body mass, health status and fitness level of the participants in the present paper. Readers should not have to retrieve this information from previous publications.

No clear rationale is provided for the four selected exercise protocols and it would be nice if the protocol selection became more apparent to the reader based on the rationale presented in the introduction.

Please be consistent with the reference to time points. E.g. T1 is referred to as “at the end of cycling” (L163), “directly at the end of exercise” (L248), and elsewhere simply “at the end of exercise”. Using either “at T1” or “immediately after exercise” would probably suffice throughout.

L126 – “and ~~at~~ **on** test days ~~themselves~~”

L129 – how were subjects selected? Please detail.

L133 – “felled back” is not correct English, suggest “and pedal frequency dropped/decreased below 70 RPM”.

L136 – Please briefly explain the rationale for the semi-crossover design, was something determined at rested baseline? Additionally, rather than using “protocol 1-5” it might be more intuitive for the reader to follow if you were to describe the exercise protocols by the specific variables that are being manipulated between protocols.

L144 – was body mass measured nude throughout?

L148 – how was baseline hydration status (pre-exercise) determined after one day of prior fluid restriction?

L152 – is it possible to provide a reference for 200ml tap water per hour to maintain hydration status?

L159 – “collected at” 🡪 “collected from”.

L174-183; L189-196; L200-208; L212-220 – I suggest the assay details could be left out, as they should be available in a kit insert. It would be more helpful to provide information about whether samples were analysed in single, duplicate, triplicate, and provide within and between plate CVs if available. Were all samples in range? I would also suggest to retain the dilution factors for the samples. Please also provide kit IDs and full supplier details where appropriate.

L242 – please define the “primary comparisons”

L244 – can you provide more details about the missing values? Were they randomly or systematically missing or “unquantified knowns” (i.e. samples below assay detection limits)?

**Results**

The results contain no p values or quantitative statements in-text. This prevents proper scrutiny of the statistical analysis and interpretation of the results. Please consider supporting statements with statistics and other quantitative statements as well as references to appropriate figures and tables.

No data are provided about the exercise protocols. Did the participants respond as expected? What was measured to ensure the protocols were delivered correctly – have you any measures of e.g. heart rate, metabolic rate, substrate utilization, power output, perceived exertion? How did these differ between the protocols?

Please also report the mean and variance of achieved dehydration status of the participants in the dehydration exercise trial. From Kartaram et al (2020), it appears that body mass loss during exercise was 1.4% (difference between trials c. 1%) with no notable change in body mass resulting from the pre-exercise fluid restriction. I would argue that this is at the minimum limit to describe a dehydration intervention (1% difference between trials) – and should at best be referred to as mild dehydration as in the previous paper.

L248 – Salivary “levels” of SLPI – is this a quantified concentration and if so, I suggest you use “concentration” with appropriate units.

**Discussion**

The authors discuss the lack of validation of salivary biomarkers. I recommend the authors consider incorporating evaluation of/discussion of biological variability statistics used to evaluate the utility of candidate biomarkers in clinical chemistry for both population cut-off and biological monitoring applications. See Cheuvront et al (2010) Am J Clin Nutr for an example of application of these statistics in a sports science/hydration context.

The authors mention that they could not obtain flow rates, but did the authors consider correcting for e.g. total protein or osmolarity in the saliva samples to account for artefacts created by e.g. changes in saliva flow rate during dehydration or after exercise?

It would be helpful if the authors could justify the choice to not include any known immune responses to exercise for comparison in the present study. How is one supposed to evaluate the response of novel immune markers to relatively novel protocols without a benchmark series of biomarkers that would be expected to respond based on current understanding? Would it be possible to incorporate previous results from the same study protocol in the discussion, for example?

Lactoferrin only responded significantly to the dehydrated condition. Could this be an artefact of salivary flow rate leading to an acute concentration effect purely due to fluid losses from saliva secretions?

IL-6 is described as a myokine produced in the muscle and tends to produce a robust response to most types of exercise in plasma/serum. Was IL-6 measured in serum/plasma in the present study? Given the authors cite a study showing low correlation between serum and saliva responses, and that the IL-6 response in saliva in the present study was relatively low, could the authors evaluate the likely utility of measuring a marker produced by exercising muscle in saliva? In short - could saliva as a matrix too far removed for measurement of exercise-induced IL-6 responses?

The paragraph on dehydration (from L339) seems relatively far removed from discussion of the findings of the present study in context. At L364 a study is cited that found dehydration did not impair salivary AMP responses to endurance exercise. At L366 the authors speculate that dehydration would drive salivary biomarker concentrations higher. Did you expect that the protocol following fluid restriction would exacerbate biomarker responses and if so, based on a “true” stressor on the immune system or as an artefact of fluid balance shifts?

The discussion lacks a full evaluation of strengths and weaknesses of the study design.

L274 – This statement that SLPI and MMP-9 warrant further investigation as biomarkers of exercise effects on immune function requires further justification. Why is this the case? How do the data enable you to conclude this?

L277 – It needs to be clearer in the results section how the responses of SLPI and MMP-9 “relate to the workload”. Workload in the protocols was not incremental but involved manipulation of multiple distinct prescription factors such as hydration, intensity and continuous/intermittent exercise. Please consider how this “relationship” could be statistically demonstrated from your data.

L278 – does blood collection always require a trained professional (e.g. if collecting capillary samples – which can also be done in the field?)

L282 – If the authors have 5 repeated baselines measured under controlled conditions, would this give additional useful information when assessing the biomarkers’ suitability for diagnostics/monitoring applications?

L284 – please explain the jump from previously demonstrated immune responses to exercise to the need/attraction of standardised exercise models.

L291 – please consider how your data demonstrate this “relationship” between workload and SLPI and MMP-9 concentrations.

L295 – it is unclear how absolute higher concentrations in saliva indicate that SLPI is an “early stress marker”, please explain.

L315 – sentence needs a reference – which cells and mechanisms?

L325 – how do you determine that these markers were affected to a lesser extent?

L357 – flow rate not possible, but correction for osmolarity or total protein would have been?

L368 – is blood osmolarity more tightly homeostatically regulated than e.g. saliva composition?

L369 – is this statement a hypothesis, a result, or a question for the future?

Conclusion

It is not clear from the discussion how the observations of biomarker responses to exercise in various exercise protocols in the present study lead the authors to conclude that the markers have potential. It could therefore be helpful to define the criteria or expected results for a biomarker that has potential a priori, based on a logical rationale and/or associations with other outcomes, to enable this conclusion later.

L374 – suggest that the applications are either taken in the discussion

L376 – this statement is a nice summary but could do with more mechanistic support from the discussion.

**Tables**

The caption of Table 1 displays “model statistics” but actually only raw and adjusted p values are given. It is not clear what the p values in the table represent. If only overall model fit, where are the p values for the main effects and interactions?

Is it possible to add concentration data to Table 1 or another table as appropriate?

**Figures**

Significantly different pairwise comparisons between protocols and timepoints do not appear to be represented on the figures, which is a shame. Please consider including these graphically or at the very least in the figure captions.

Figure 1b appears surplus – it is appropriate to consider that absolute concentrations vary between sample matrices.

If it would be possible to provide a y axis with back-transformed raw concentrations on a log scale, this would aid interpretation in terms of normative values for the reader.

**Data availability**

I tried to access the link provided to the R code/statistical analyses and the link appeared dead. Please check this!
