# Supplementary material for: Salivary concentrations of secretory leukocyte protease inhibitor and matrix metallopeptidase-9 following a single bout of exercise are associated with intensity and hydration status
Source: PLoS One. 2023 Nov 22;18(11):e0291297. doi: 10.1371/journal.pone.0291297 (PMC10664895; doi:10.1371/journal.pone.0291297)
Supplement: Attachment — Submitted filename: Rebuttal PONE-D-22-17078R1.docx [file pone.0291297.s003.docx]

Review Comments to the Author

Reviewer #3: The Authors extensively answered on the Reviewers' comments which were very constructive. Only some minor typographical errors in the text and in the Table 1 need correction. **The authors would like to thank again the reviewer for his thorough review which has improved the quality of the manuscript significantly. We have reviewed the paper for typo’s and hope to have all captured**

Reviewer #4: The present revised manuscript describes the results of a study investigating the alteration of different immune marker in salvia with specific focus to SLPI and MMP-9 under different exercise conditions and hydration status. Beside oft hem the alteration of immune marker in the blood are shown. The study seems in principal well done and presented. The discussion and conclusion are a bit speculative. The relevance of the findings for exercise immunology isn’t fully clear. Nevertheless the findings are interesting and give new information about the alteration of specific immune marker in salvia. Limitations are given in the specific comments. **The authors would like to thank again the reviewer for his thorough review which has improved the quality of the manuscript significantly**

Specific comments:

1. From the present data it is difficult to conclude that SLPI and MMP-9 could be a general marker for immune response or it is only a marker for mucosa immune response. This should be pointed out. **The authors agree that these markers are not general/mucosal immune markers, since they both have anti-inflammatory actions, in the abstract & overall conclusion ‘immune responses’ has been replaced with ‘(local) inflammatory responses’.**

2. It would be helpful to show direct correlations of the salvia and serum level. **We have studied the direct correlations (per timepoint) of the markers. The difficulty in this lies in the enormous difference in concentration between serum and saliva (which is why the graphs are presented as log-transformed), especially for SLPI which is extremely low in serum. Also, MMP-9 levels in saliva do not directly reflect blood concentrations since the pattern of increase is different in saliva from that in plasma (this sentence is mentioned in the discussion). For these reasons we decided that it did not contribute to the message of the paper and left it out. However, all data will be available for everyone who would like to perform other calculations with the data.**

3. In the figures T2, T3 are better given. Significant values should be labelled in the figures. **Significance (*) has been added to the figures**
